# The impact of tree age on biomass growth and carbon accumulation capacity: A retrospective analysis using tree ring data of three tropical tree species grown in natural forests of Suriname

**DOI:** 10.1371/journal.pone.0181187

**Published:** 2017-08-16

**Authors:** Michael Köhl, Prem R. Neupane, Neda Lotfiomran

**Affiliations:** Center for Earth System Research and Sustainability, World Forestry, Universität Hamburg, Hamburg, Germany; INRA - University of Bordeaux, FRANCE

## Abstract

The world’s forests play a pivotal role in the mitigation of global climate change. By photosynthesis they remove CO_2_ from the atmosphere and store carbon in their biomass. While old trees are generally acknowledged for a long carbon residence time, there is no consensus on their contribution to carbon accumulation due to a lack of long-term individual tree data. Tree ring analyses, which use anatomical differences in the annual formation of wood for dating growth zones, are a retrospective approach that provides growth patterns of individual trees over their entire lifetime. We developed time series of diameter growth and related annual carbon accumulation for 61 trees of the species *Cedrela odorata* L. (Meliacea), *Hymenaea courbaril* L. (Fabacea) and *Goupia glabra* Aubl. (Goupiacea). The trees grew in unmanaged tropical wet-forests of Suriname and reached ages from 84 to 255 years. Most of the trees show positive trends of diameter growth and carbon accumulation over time. For some trees we observed fluctuating growth—periods of lower growth alternate with periods of increased growth. In the last quarter of their lifetime trees accumulate on average between 39 percent (*C*. *odorata*) and 50 percent (*G*. *glabra*) of their final carbon stock. This suggests that old-growth trees in tropical forests do not only contribute to carbon stocks by long carbon resistance times, but maintain high rates of carbon accumulation at later stages of their life time.

## Introduction

Close to 90 percent of the global terrestrial biomass carbon is tied up in forests, of which almost half is contributed by tropical and subtropical forests. [1] In the period from 2011 to 2015 the average annual net removals by forests reached -0.57 Gt C globally. By contrast, in the same period annual emissions from deforestation and forest degradation, which to a large extent took place in tropical and sub-tropical forests, reached 0.8 Gt C and 0.27 Gt C, respectively [2].

With regard to carbon accumulation the growth pattern of old trees is of particular importance [3 4]. The prevailing opinion arising from traditional growth and yield studies in managed single-species, even-aged forest stands stipulates decreasing height, diameter and volume growth at older tree ages, resulting in a sigmoid shaped growth curve [5, 6]. Declining tree growth over time is attributed to variations in the supply rate of required resources (light, nutrients, water), changing balance between photosynthesis and respiration, increased hydraulic resistance, decreased nutrient supply, or genetic changes with meristem age [7–12]. Delzon et al. [13] report a height-related decrease in above-ground annual biomass increment per unit leaf area. Recent studies highlight sustained or continuously increasing mass growth rates with increasing tree size and emphasize the significant role of old trees for carbon accumulation [14–16]. Among others the following reasons for sustained tree growth with age were mentioned: (1) the metabolic scaling theory (MST), which stipulates a continuously increasing mass productivity with tree size [17, 18], (2) the competition for space [19], (3) the increase of a tree’s total leaf area and positive feedbacks of light environment with tree growth [14, 20, 21], or (4) the process of adaptive reiteration (AR), which decreases the ratio of respiration to photosynthesis, rejuvenates apical meristems, and improves the hydraulic conductance by newly developed leaves [22]. In a recent review [23] critically analyzed studies on biomass growth increases in large trees. The physiological underpinnings of growth patterns of large and presumably old trees remain poorly documented. As a consequence it is not surprising that we have little knowledge on aging processes of old trees and their relation to tree growth.

Statements reflecting the growth of old trees and associated carbon accumulation are generally based on data obtained from long-term forest monitoring plots. Due to the longevity of trees the available data of long-term monitoring plots frequently cover only a limited time section of the total lifespan of trees. A complement to long-term monitoring is offered by tree ring dating (dendrochronology), which studies the growth of individual trees by analyzing their pattern of growth rings caused by annualities in wood formation. Annual rings are formed in trees where the climate halts growth at some point in the year. This results in anatomical differences in wood formation. In temperate and boreal climates the formation of tree rings is caused by seasonal changes of temperature. In tropical and sub-tropical regions short annual dry seasons with less than 60 mm of monthly rainfall or regular flooding that lead to anoxic conditions in the root space and following water deficit in the crown induce cambial dormancy [24].

Dendrochronology in the tropics exists since more than one hundred years [25]. The existence of annual tree ring growth has been described and verified for numerous tropical tree species [26–36]. However, due to their large variety many tree species have not yet been studied. Despite the fact that tree ring analysis for tropical trees is not straightforward, tropical tree ring analysis made substantial progress during last two decades [37]. Its potential to provide valuable information on individual age and growth pattern as well as dynamics in tropical forest is well know [38–41].

The aim of this study is to describe the growth pattern of individual trees over their entire lifetime using tree ring analyses and to derive verifiable statements on diameter growth and related carbon accumulation rates at advanced tree ages. We analyzed the individual tree growth patterns of 61 trees, which grew without any human intervention in natural forests of Suriname. The diameters were transferred to aboveground biomass (AGB) by allometric equations [42]. Following [43] AGB was converted to carbon content.

Our retrospective analysis, which covers the entire lifespan of trees, adds new evidence for sustained growth and carbon accumulation by old trees. In terms of the growth and carbon accumulation patterns, we aim firstly to describe growth patterns and associated C-accumulation of old trees. We then review findings of recent studies on the significant role of old trees for carbon accumulation. Finally we compare declining growth patterns observed in managed forest with the continuing strong growth patterns we observed in natural forests and present an explanatory approach to solve the contradictory implications for forest management.

## Materials and methods

### Tree origin

**Fig 1:**
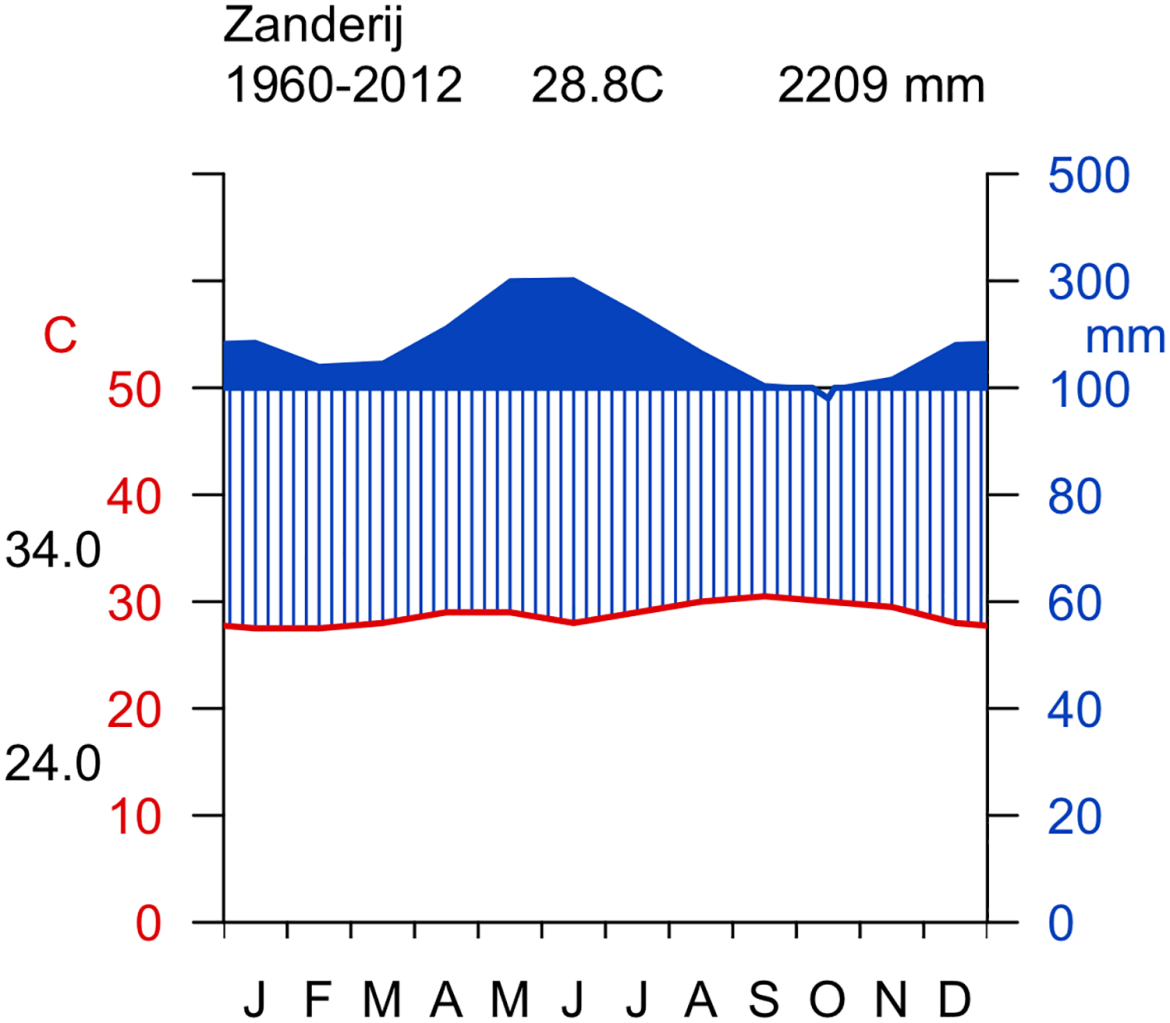
Climate diagram for metrological station in Zanderij. The climate diagram offers to distinguish four seasons. The main rainy season extends from April to August and is followed by the main dry season from September to November. The average annual temperature is 27.5°C with an annual variation of 3°C. The diagram was created by means of the R package Climatol [47].

The trees selected for the study grew in the central part of Suriname’s forest belt. Suriname is a tropical country located in the northeast of South America near the equator. About 95% (153,320 km^2^) of the total land area is forested [44]. Most of the tropical rain forest occurs in the hilly and mountainous interior of the country. Approximately 660 tree species grow in Suriname’s forests, of which more than 200 species are currently used commercially [45]. Suriname has a tropical climate with an average annual temperature of 27.5°C and annual variation of only 3°C and a daily range between 23 and 33°C. The average annual rainfall is about 2,200 mm with deviations across the country from 1,500 mm on the coast to 2,500 mm in the higher areas in the central and southern regions [46]. The climate diagram offers to distinguish four seasons (Fig 1). The main dry season, which occurs consistently each year from September to November, is likely to cause the annuality of wood formation [24].

### Tree selection

For our study we selected trees of the three species *Cedrela odorata* L. (Cuban cedar, family: Meliacea), *Hymenaea courbaril* L. (Rode lokus, Jatobá, caca chien, family: Fabaceae), and *Goupia glabra* Aubl. (Kopi, family: Goupiaceae), which are trees of the New World tropics. The selected species show anatomical characteristics that allow for identifying tree-ring boundaries. No trees were explicitly felled for our study. All trees utilized were cut for legal commercial harvests. In Suriname approximately 15000 trees of the species *C*. *odorata*, *H*. *courbaril*, and *G*. *glabra* were legally harvested in 2014 to 2016 according to Suriname’s national record of logged trees. From these trees we randomly selected a sample of 61 trees, which includes 20 trees for each of the species *C*. *odorata* and *H*. *courbaril* and 21 trees of the species *G*. *glabra*.

The trees grew without any human intervention in natural forests of Suriname. *C*. *odorata* is known as a “gap opportunist” and reaches dominant or co-dominant positions in the crown canopy under adequate light conditions [48]. *G*. *glabra* is a shade-intolerant pioneer species and has comparatively high growth rates if exposed to direct sunlight [49, 50]. *H*. *courbaril* tolerates poor fertility and extended periods of droughts, but is intolerant to shade at mature ages [51, 52]. They are therefore prone to feedbacks of light environment with tree growth. We selected *C*. *odorata*, *H*. *courbaril*, and *G*. *glabra* due to the clear visibility of growth rings [41]. The annuality of wood formation is caused by Suriname’s climate with a main dry season from September to November. From the randomly selected trees we extracted cross-sectional wood discs at the lower (i.e. thicker) end of the logs. With a legal approval issued by the Suriname’s Foundation for Forest Management and Production Control (SBB) the discs were shipped to Hamburg, Germany, for further analyses.

### Tree ring methods

**Fig 2:**
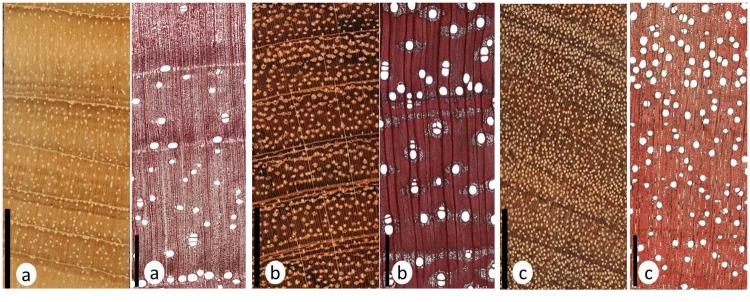
Macro (right)—and micrographs (left) of *C*. *odorata* (a), *H*. *courbaril* (b), *G*. *glabra* (c). Scale bar for macrography = 1cm, for microscopic cross sections = 500 μm.

Anatomical characteristics of tree-ring boundaries were investigated by means of microtome sections with a thickness of approximately 20 μm. The microtome sections were stained with safranine to enhance the contrast between vessels, fibers and parenchyma cells of the various wood tissues and provided anatomical characteristics for identifying tree ring boundaries. Fig 2 presents macro and micro-sections of *C*. *odorata*, *H*. *courbaril*, and *G*. *glabra* species and distinctiveness of tree ring boundaries. The annual nature of tree rings in *C*. *odorata*, *H*. *courbaril* and *G*. *glabra* has been verified by previous studies in Suriname as well as in other South American regions [38, 53–55]. The anatomical features of the three tree species are described by [41].

The stem discs selected were air-dried and polished with sandpaper (up to 1000 grain). On each disc clearly visible rings easily traceable were marked across the cross-section starting from the pith (center) to the periphery. They served as a chronological skeleton for validating tree-rings in disc surface compartments where the growth-ring structure was poorly visible. At least 4 radii were selected for measuring the width of tree rings. In total 369 radii were measured. Wedge and discontinuous rings, which occur only over a part of the disc, were identified to avoid errors in ring marking. For trees with several multiple wedging rings, it is good practice to choose those radii, which correspond best to the average diameter of the disc [56]. Tree ring boundaries were marked under a binocular and every tenth ring was interconnected between radii to verify the correct marking.

The width of the increment zones was measured to the nearest 0.01 mm along at least eight disk radii using a moving table (LinTab Measuring System, RinnTech Heidelberg, Germany) connected to a PC. The measurements were plotted as increment curves. Subsequently the tree-ring series were cross-dated first within individual trees and then among all trees. This allowed for synchronizing similar characteristics of growth patterns of individual trees and for assigning tree rings to distinct years of wood formation. From the tree-ring series along the radii, a mean tree-ring series was calculated for each tree [56, 57].

### Estimating tree biomass and annual carbon accumulation

The aboveground biomass was estimated by a published allometric equation that relates diameter (D), wood density (**ρ**) and an environmental stress factor (E) to the weight of aboveground biomass (AGB) [58]. We used the following equation, which was developed for trees from a pantropical compilation that performed well for a French Guiana dataset. This region is adjacent to Suriname and has the similar forest types and climatic conditions:

$$AGB_{est}=exp\left\{-1.803\;–\;0.976\text{ E }+\;0.976\text{ ln}\left(\rho \right)+\;2.673\text{ ln}\left(D\right)-0.0339{\left[ln\left(D\right)\right]}^{2}\right\}$$ (1)

This equation was proposed for the situation in which height measurements are not available. The environmental stress factor E increases with the seasonality of temperature and the time when plant is exposed to water stress. It was calculated by the R-routines made available by [58]. Wood densities (**ρ)**were taken from Comvalius (45) and [59] (*C*. *odorata* **ρ** = 0.38 g cm^-3^; *H*. *courbaril* **ρ** = 0.77 g cm^-3^, *G*. *glabra* **ρ** = 0.72 g cm^-3^) and utilized to convert biomass weight to carbon mass. Annual C-accumulation rates were derived from the difference of total carbon content between two consecutive years.

Eq (1) assumes that diameters are taken at the trunk base of a tree. This is typically the height above ground at which felling cuts are applied, unless a tree develops strong, supporting root systems. For *C*. *odorata* buttresses are absent or small, so that there are reasonable grounds to assume that diameters at the lower end of the log and diameters at trunk base are similar [52]. However, as trunk diameters are generally larger than diameters in upper stem heights, our AGB and carbon estimates are conservative and do not overestimate true values.

### Time series analysis

Monotonic trends in time series analyses can be detected by either parametric or non-parametric procedures. As parametric procedures make strong assumptions for the distribution of data, which are difficult to verify and often not fulfilled, we choose the non-parametric Cox-Stuart trend test [60] to test our time series change trend. For testing the series for a shift of the central tendency Pettitt’s test was applied [61]. It utilizes a nonparametric U-test to test for a shift in the central tendency of a time series, i.e. variables follow one or more distributions that have the same location parameter (i.e. no change), against the alternative that a change point exists. We used the R package TREND to perform the Cox-Stuart trend test and the Pettitt’s test [62, 63].

For each tree the entire C-accumulation series was divided into 4 periods of equal time intervals and the mean C-accumulation calculated for each interval. For each tree the mean C-accumulation rates between the four quarters of life time were tested for differences with Dunn’s test for multiple, pairwise comparisons [64] using the R package “dunn.test” [65]. The R-package “Forecast” was utilized for plotting time series and calculating moving averages [66].

## Results

### Age, diameter and accumulated carbon stock at end of lifetime

The age of the trees included in our sample ranges from 84 years to 255 years and stem diameters range from 36.7cm to 99.2 cm. The individual tree carbon stock accumulated at the time of harvest ranges between 329 kg and 7319 kg (Table 1). ANOVA showed significant differences (p = 0.0002) between species for diameter and thus for accumulated C-stock estimate. Pairwise comparisons using t-tests with pooled SD and p-value adjustment according to Holm [67] identified significant differences for diameter between *H*. *courbaril* and *C*. *odorata* and *H*. *courbaril* and *G*. *glabra*, and for accumulated C-stock between all paired combinations of *H*. *courbaril*, *G*. *glabra* and *C*. *odorata*.

**Table 1:** Age, diameter and C-stock by tree species.

Species	No. of trees		Age	Diameter	Carbon-stock
			[years]	[cm]	[kg]
*C*. *odorata*	20	Mean	138	52.3	819
		Min/ Max	84/180	36.7/ 64.9	329/ 1320
*G*. *glabra*	21	Mean	149	54.4	1685
		Min/ Max	112/189	40.3/ 75.8	772/3523
*H*. *courbaril*	20	Mean	155	67.8	3157
		Min/ Max	87/ 255	40.8/ 99.2	849/ 7319

**Fig 3:**
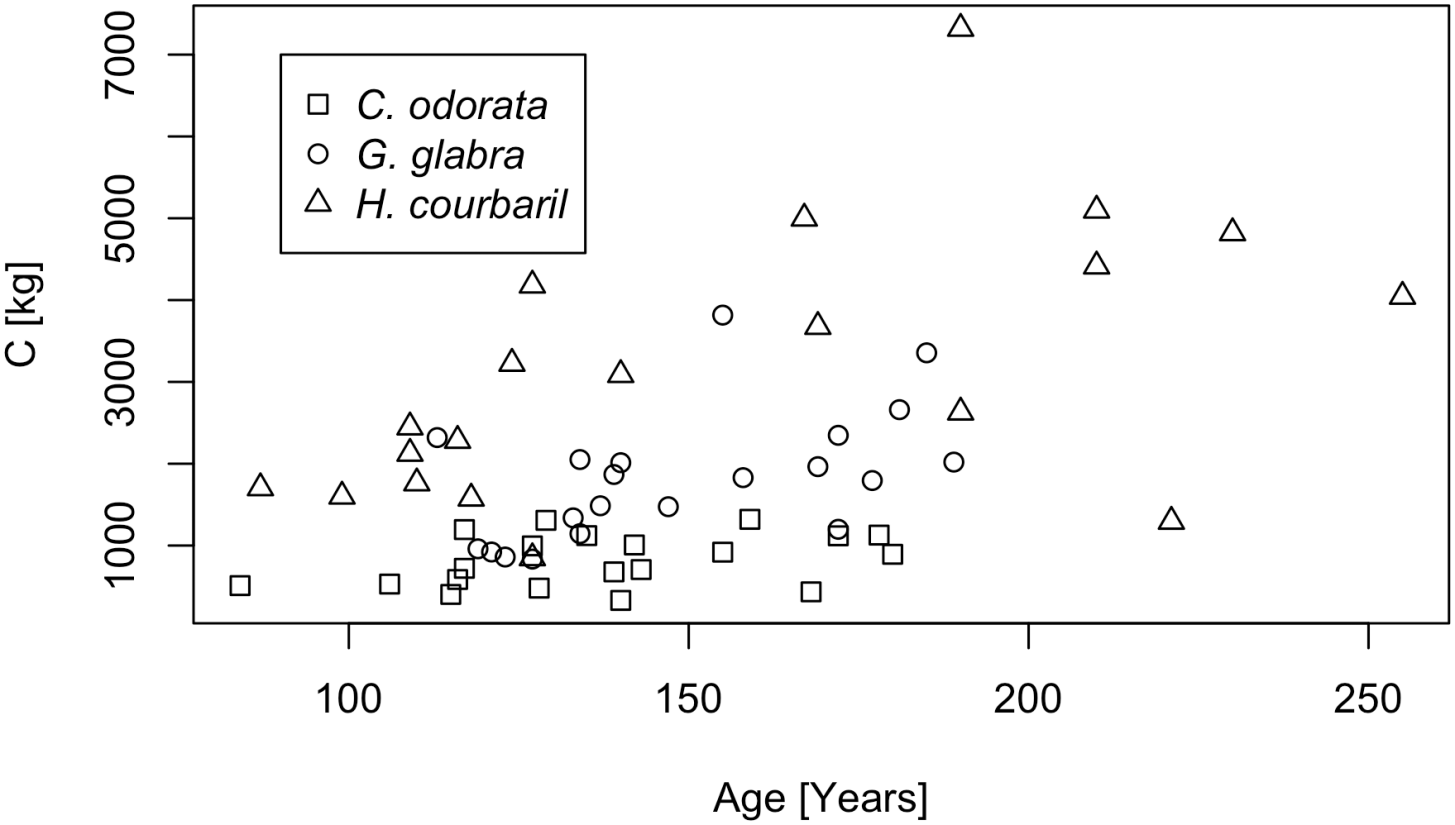
Carbon stock over tree age. The figure presents the carbon stock accumulated at the end of the lifetime for the 61 trees included in the study. Different colors and symbols present tree species. For *C*. *odorata* the individual tree carbon stock does not increase with age. For *G*. *glabra* larger carbon stocks are found for older ages, but some trees accumulated relatively large carbon stocks even at younger ages. Except for two outliers only for *H*. *courbaril* carbon stocks increase with age, but still show a considerable variability.

Fig 3 presents the relationship between tree age and accumulated carbon stock for the three species. For the entirety of the 61 studied trees the Pearson's product-moment correlation is significant (r^2^ = 0.26, p = 2.954e-05). The correlation is smaller for *G*. *glabra* (r^2^ = 0.28, p = 0.01438) than for *H*. *courbaril* (r^2^ = 0.31, p = 0.01068). No significant correlation was found for *C*. *odorata*. The relatively low correlation coefficients indicate that for the trees in our sample, age alone is not decisive for the amount of C accumulated at the end of their lifetime.

### Annual tree related carbon accumulation

Table 2 presents the mean annual C-accumulation, which was smallest for *C*. *odorata* and largest for *H*. *courbaril*. This partly reflects the differences in specific wood gravity. Mean annual tree accumulation was found to be significant (**α** = 0.05) between all pairwise combinations of the species.

**Table 2:** Annual carbon accumulation and annual diameter growth of trees by species.

Attribute		Species		
		*C*. *odorata*	*G*. *glabra*	*H*. *courbaril*
Annual C-accumulation [kg]	Mean	6.0	12.2	20.3
	Min/Max	0.0/44.1	0.0/186.1	0.0/ 215.3
	SD	6.1	13.2	21.6
Annual diameter growth [cm]	Mean	0.38	0.37	0.44
	Min/Max	0.04/2.51	0.11/2.64	0.04/1.98
	SD	0.28	0.17	0.29

SD: standard deviation; Min: minimum; Max: maximum.

**Fig 4:**
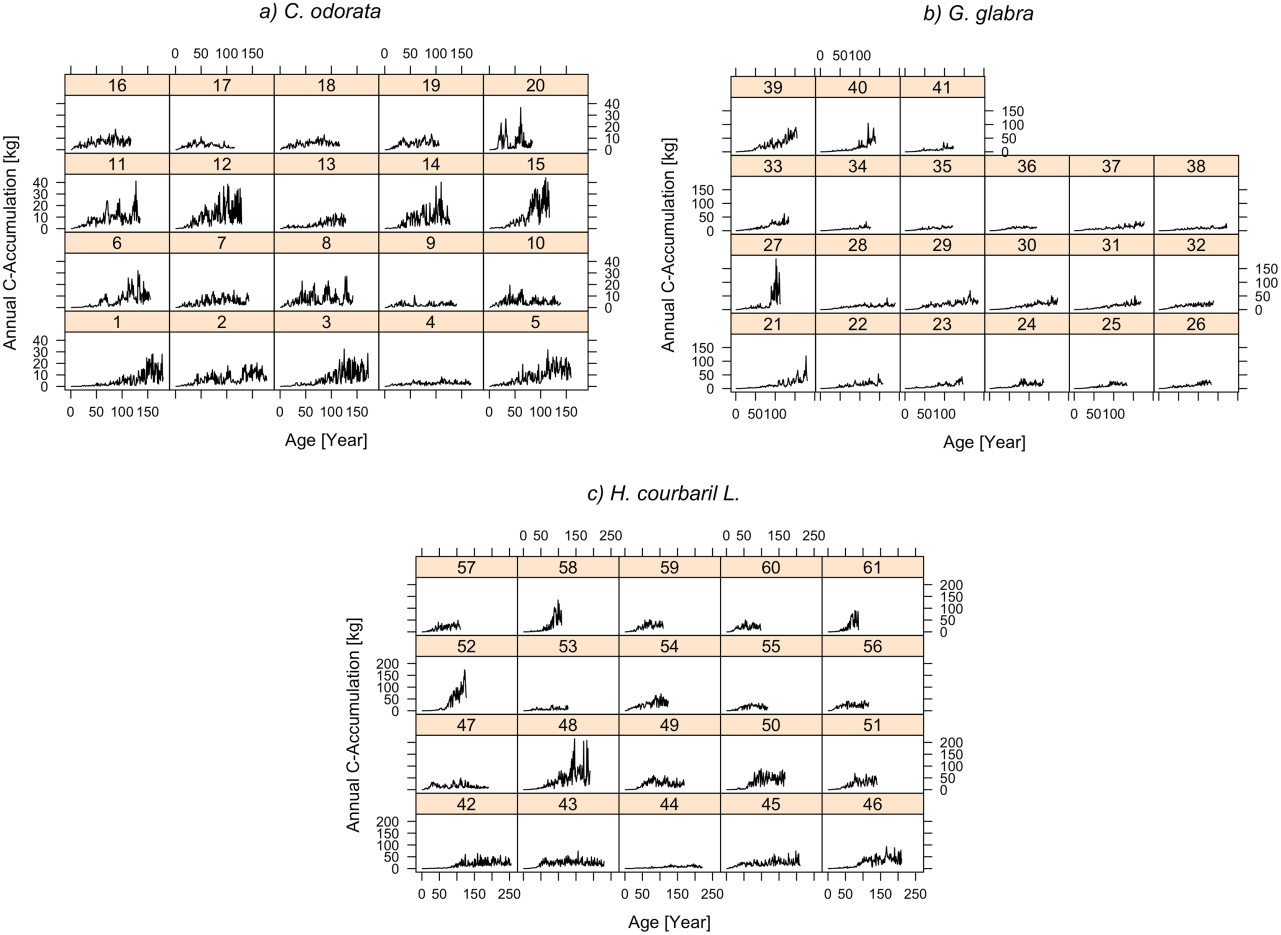
Annual C-accumulation. a) *C*. *odorata*, b) *G*. *glabra*, c) *H*. *courbaril* For individual trees the annual C-accumulation is plotted over tree age. Figures above the curves indicate the tree number. Plots are grouped by tree species. The scale of the y-axis varies between tree species, i.e. {0–40} for *C*. *odorata*, {0–150} for *G*. *glabra*, and {0–200} for *H*. *courbaril*.

**Fig 5:**
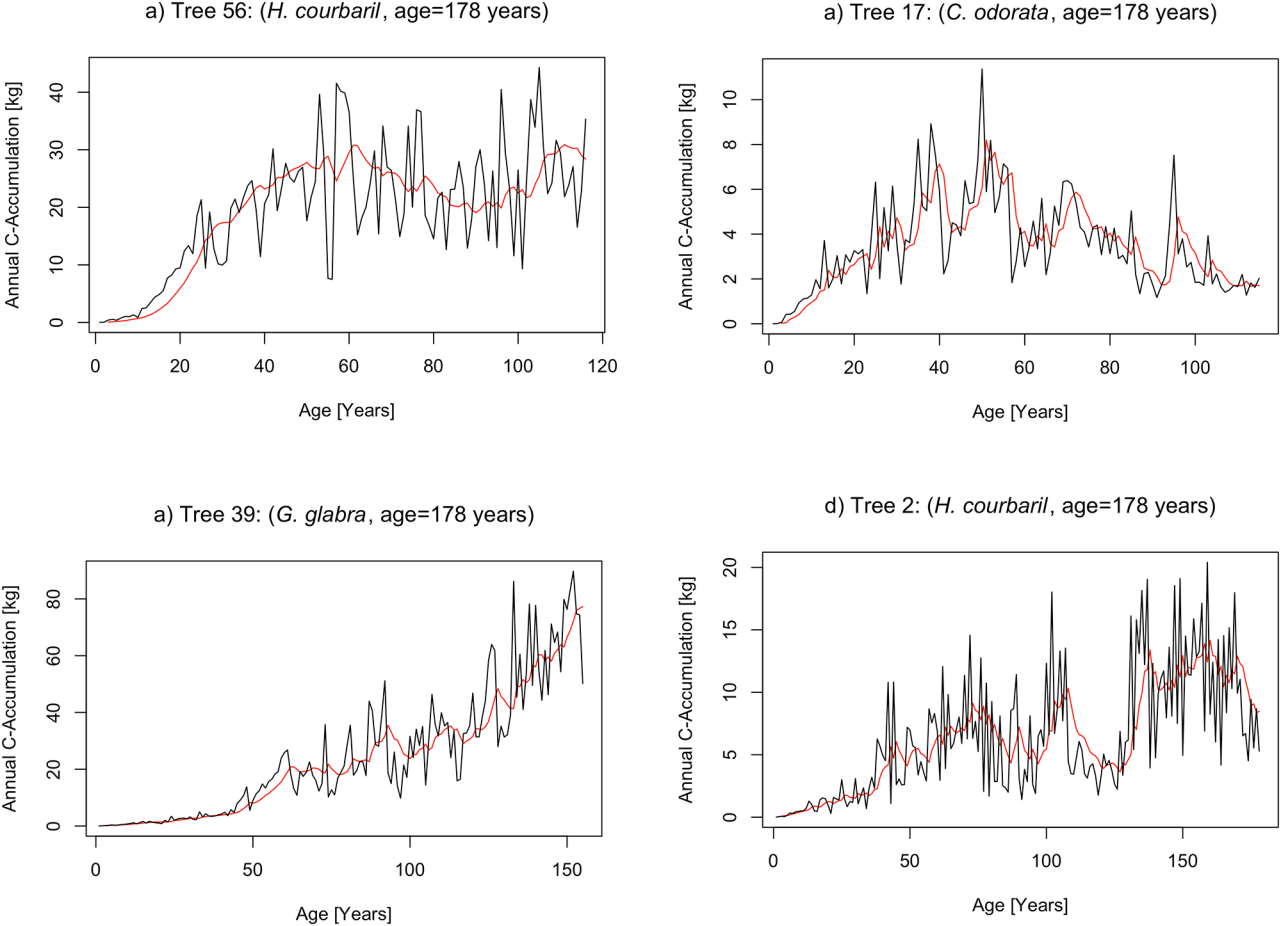
Examples for different annual patterns of C-accumulation over tree age. Black lines show the annual C-accumulation, red lines present moving averages.

Across the 61 trees, annual C-accumulation over age is displayed in Fig 4. Noticeable are the explicit differences in annual C-accumulation between adjacent years. For all trees annual C-accumulation is characterized by low rates in young ages followed by increasing rates at older ages. This is partially due to the fact that we used diameters (D) to calculate AGB, which enter squared in Eq (1). However, the 61 trees observed did not show a uniform pattern of annual C-accumulation over time, but allowed to identify four general growth patterns, which are shown in Fig 5. Tree 39 maintains an increase of C-accumulation with increasing age. The same holds for Tree 2, but with a clear depression between an age period from 110 to 140 years. Tree 56 shows a C-accumulation pattern that is characterized by relatively uniform rates after an initial phase and shows no trend. In turn the accumulation pattern of tree 17 exhibits an increase in the first half of life followed by a continuous decrease that is maintained until its end of life.

### Total carbon accumulation of individual trees

**Fig 6:**
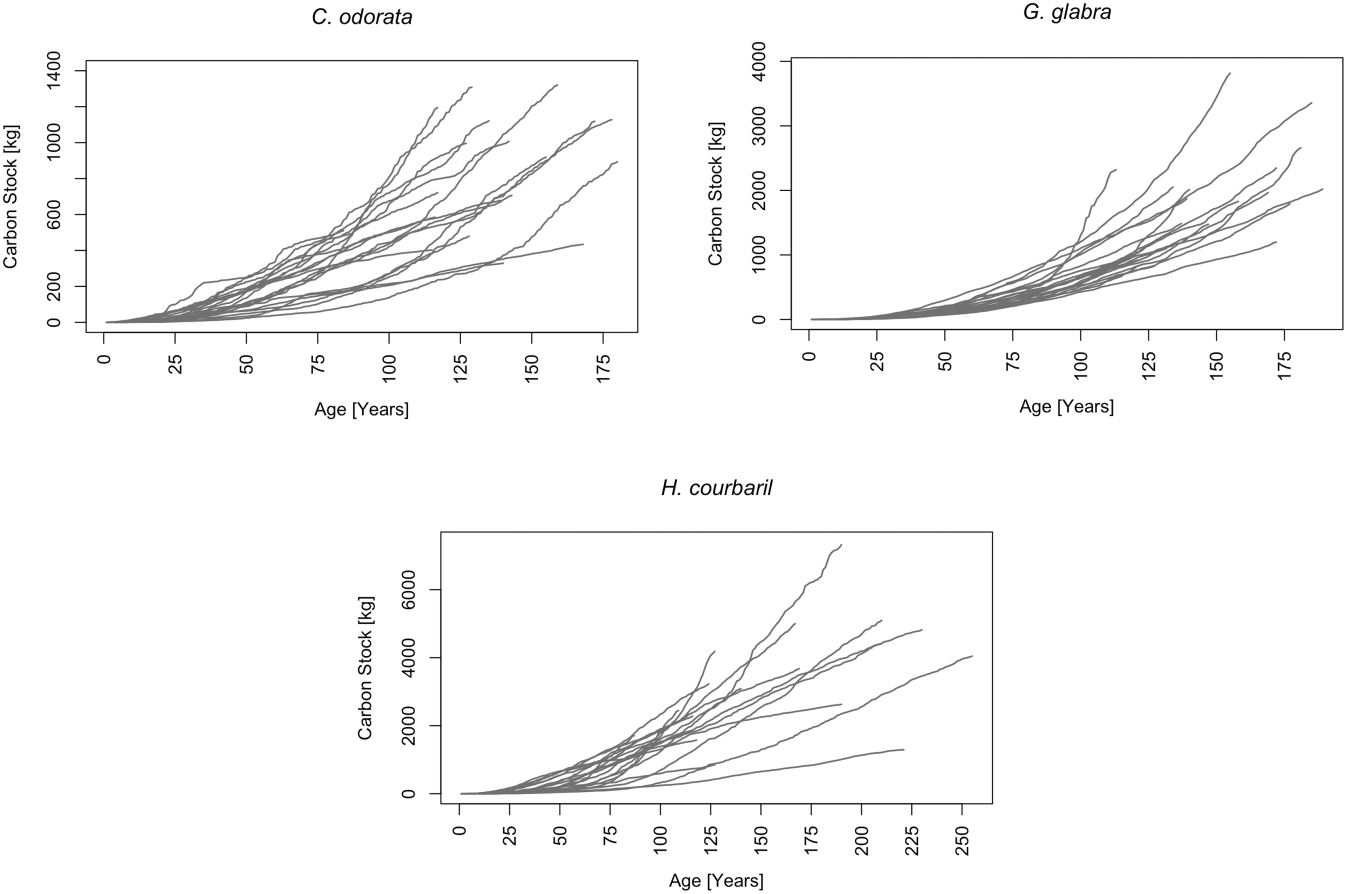
Individual tree C-accumulation. a) *C*. *odorata*, b) *G*. *glabra*, c) *H*. *courbaril* The curves show the development of the carbon stock of individual trees over age. Most curves approach the shape of an exponential function, which indicates accelerated C-accumulation with age. In the few cases where curves become flatter, trees reduce carbon accumulation with age.

We transformed the annual C-accumulation rates to cumulative growth curves, which describe the development of individual tree C-stock over time. In managed, even-aged forest stands growth curves are characteristically S- or sigmoid-shaped and show distinct periods of youth, maturity and senescence. During youth the growth rate increases rapidly to a maximum at the point of inflection and decreases further onwards [6, 19]. In Fig 6 the growth curves for the 61 trees included in our study are presented. It is obvious that tree growth does not follow a sigmoid-shaped curve. Trees either maintain a relatively constant growth rate or show increased growth at older ages. For 57 out of the 61 trees this trend is significant, for four trees (Trees number 10, 17, 20, and 47) no significant trend could be observed (Cox-Stuart trend test, **α** = 0.05) [60].

### Development of individual tree carbon accumulation over lifetime

In order to illustrate the development of C-accumulation over time, each tree’s entire lifetime was separated into four time periods of equal length. Table 3 presents the percent C-accumulation for the respective quartiles of lifetime. The mean C-accumulation increased consistently with lifetime. In the first quarter of lifetime the mean C-accumulation ranges between 3.6 percent (*G*. *glabra*) and 8 percent (*C*. *odorata*) and in the last quarter between 38.6 percent (*C*. *odorata*) and 50.2 percent (*G*. *glabra*). On average 69 percent (*C*. *odorata*) to 80.1 percent (*G*. *glabra*) of the total C-accumulation are accumulated in the second half of life.

**Table 3:** Percent C-accumulation by lifetime quartiles.

Quartile		*C*. *odorata*	*G*. *glabra*	*H*. *courbaril*
		[%]	[%]	[%]
1st	Mean	8.0	3.6	5.1
	Min/ Max	1.6/ 19.0	1.6/ 6.4	0.3/ 23.6
2nd	Mean	23.0	16.3	19.3
	Min/ Max	9.4/ 39.1	8.2/ 23.1	3.6/ 34.4
3rd	Mean	30.4	29.9	33.2
	Min/ Max	22.3/ 37.8	10.7/ 40.3	18.6/ 41.3
4th	Mean	38.6	50.2	42.4
	Min/ Max	18.5/ 63.5	38.9/ 77.8	18.2/ 76.8

**Fig 7:**
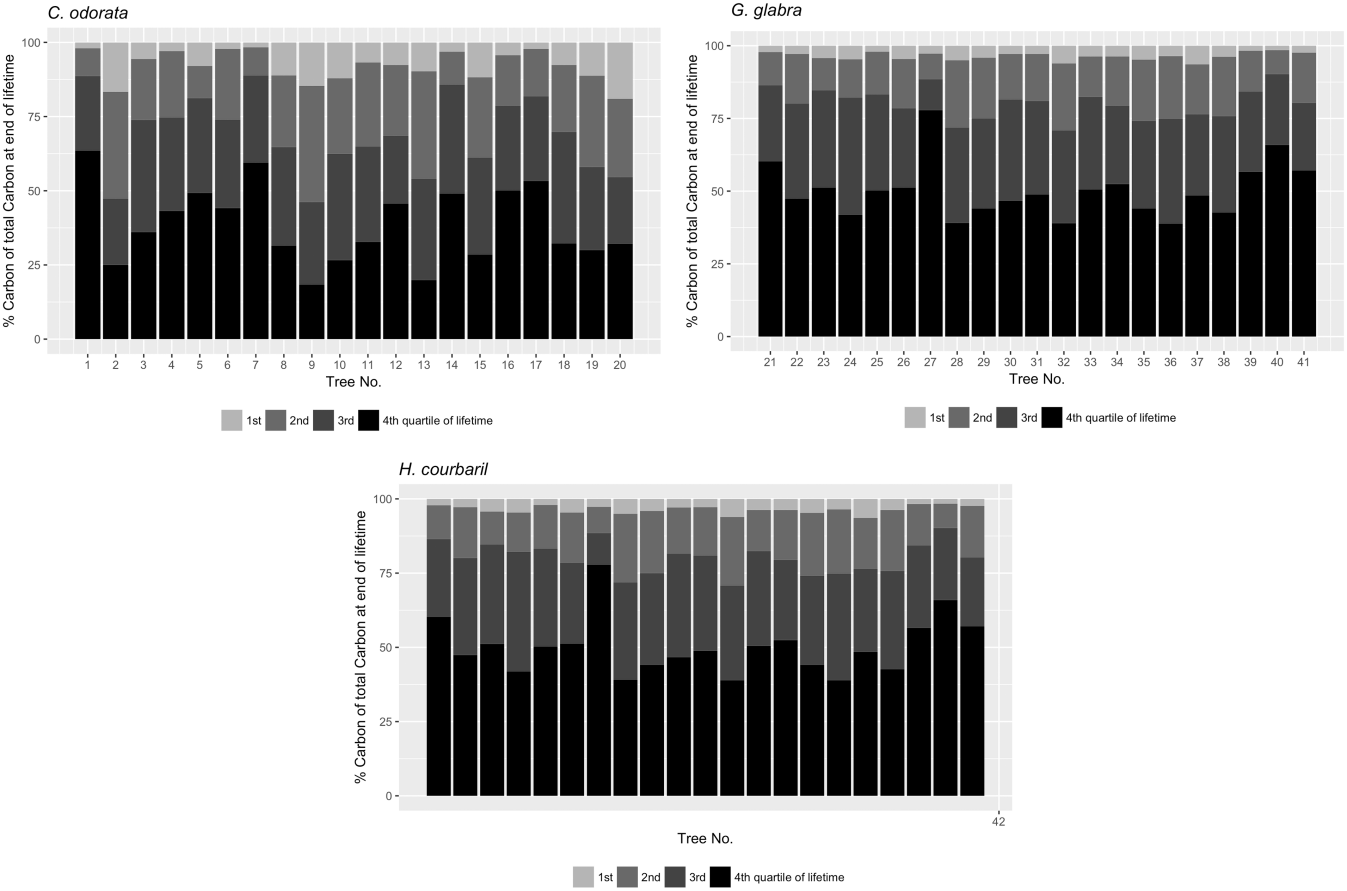
Percent C-accumulation during the four quarters of the entire lifespan by trees. a) *C*. *odorata*, b) *G*. *glabra*, c) *H*. *courbaril* For each tree the total amount of carbon sequestered in each of the four quartiles of lifetime is shown. Carbon accumulation at young ages (1^st^ quartile) is substantially lower than at older ages (2^nd^ to 4^th^ quartile). Trees of the species *G*. *glabra* sequester about half of their total carbon in the last 25% (4^th^ quartile) of their lifetime.

The C- accumulation by species for individual trees in the four quartiles is shown in Fig 7. Differences between the quartiles are significant for all species except between the 2^nd^ and 3^rd^ quartile for *C*. *odorata* and the 3^rd^ and 4^th^ quartile for *C*. *odorata* and *H*. *courbaril* (Dunn’s test for multiple pairwise comparisons, **α** = 0.05 [63–65]). This provides further evidence that C-accumulation rates of individual trees do not decline but increase at old age.

Our results show that growth and biomass accumulation of trees are subject to year-to-year and long-term variations. Over 90 percent of the trees included in our study show a positive trend, with most of the carbon being accumulated at older ages. During lifetime positive and negative trends can alternate and phases of saturation can be followed by increased growth. Consequently trees have the potential to recover from phases of saturation.

Trends and saturation phases observed for individual trees do not occur in the same calendar year. Thus, a climatic dependency on the temporal growth series cannot be assumed. Correlating annual precipitation and annual temperatures with the growth series did not show any significant relationship. It stands to reason that individual tree growth in the study area is not driven by annual climatic variations. Therefore other explanatory approaches have to be taken into consideration. One possible explanation is the dependency of tree growth and light availability, which is used in tropical silviculture to promote the growth of trees by liberating them from concurrence. As the trees included in our study were dominant or co-dominant in the crown layer, they are prone to growth reactions with respect to changes in light availability.

## Discussion

This study rests on a large dataset for three tree species of the Guiana Shield. We applied a novel approach that provides detailed insights into the carbon accumulation dynamics of individual trees over their entire lifetime. Annual diameter growth was obtained by tree ring analyses and converted into annual carbon accumulation by means of biomass functions.

### Long-term growth patterns

Tree growth in unmanaged tropical forest is prone to high variability within and between species [68–70]. Our study shows that growth and associated carbon accumulation is volatile at both, short-term and long-term time intervals. As a consequence tree age and tree size are not necessarily correlated. Carbon accumulation is comparatively low at younger tree ages. This is in line with the dynamics of tree collectives in natural forests. Small-sized trees growing in the understory need to utilize openings in the canopy and focus biomass production mainly on height growth rather diameter growth until they reach the upper crown layers [37, 69]. Carbon accumulation is large in mature trees that form the upper crown canopy. We provide additional evidence that trees can maintain high carbon accumulation rates during the latter half of their lifetime. This is in contrast to the sigmoidal growth pattern of trees cultivated in managed forests [5, 71]. Managed forests are generally even-aged, single species stands with homogeneous tree characteristics. With increasing age and tree dimensions most of the trees simultaneously reach stages of limited availability of growth factors, which explains sigmoid-shaped growth patterns and the synchrony of growth patterns of individual trees and entire stands. In a recent study [14] analyzed 403 tree species mainly assessed on permanent sample plots and conclude that biomass growth increases with tree size. The generalization of size-related growth patterns and trends observed on individual, large-sized trees is questioned critically by [23]. Due to the longevity of trees, statements on size-related tree growth are subject to the time intervals considered. The identification of general patterns in tree growth renders the reconstruction of lifetime growth necessary. Long-term growth patterns differ strongly between and within tree species [38, 72]. Our data demonstrate the volatility of annual diameter and biomass growth and underline the need for long-term growth series in order to draw conclusions on size-related growth. This holds especially true, as phases of increasing and increasing growth alter over time.

Differences in tree growth can be understood as individual tree responses to ontogenetic trajectories, competition, and site factors [68]. For Bolivian rain forests [35] related tree growth to rainfall by using tree ring analysis. They showed that growth was related to rainfall for four out of the six species studied. For *C*. *odorata* the found a high sensitivity to the rainfall well before and at the end of the growing season and the months between the previous rainy season to the beginning of the dry season. In our data phase changes from low to pronounced periods of carbon accumulation and vice versa do no occur over the same time periods and thus cannot be explained by annual variations in climate. However, the climate records available for our study are less detailed than the records available for the study by [35]. CO_2_-induced stimulation of tree growth could not be confirmed by [73, 74]. As tree size, illumination and biomass growth tend to covary in forests [23], there is evidence to suggest that the periodicity of growth patterns is driven by the competition with neighboring trees and the resulting availability of light [75, 76]. The limited availability of information on predictive variables results in a large proportion of unexplained variability of growth [68].

### Carbon accumulation and storage

Carbon stocks of forests are a result of both, carbon capture by biomass growth and the duration of carbon in biomass. Körner [77] states that “tree longevity rather than growth rate controls the carbon capital of forests”. He shows that the size of an ecosystem’s carbon pool and its carbon turnover are “commonly not related” and postulates that the carbon residence time in a system has to be extended in order to preserve carbon fluxes from the atmosphere to forest biomes. When carbon residence in a forest ecosystem is considered, the traditional perspective of sustainable forest management fails, which focuses on the balance of increment and fellings. Even when the forest ecosystem perspective is widened to a forest sector perspective the forest carbon loss induced by logging activities in tropical forests can often not be compensated by accounting for the carbon pool in harvested wood products and the carbon substitution effects by timber utilization [78]. In a recent paper [79] reported a long-term decreasing trend of carbon accumulation for Amazon forests during the past decade. They show that the decline is a consequence of shortening of carbon resistance time due to increased mortality and growth rates that level off.

Although carbon stock dynamics take place in considerably large areas, they can be attributed to the dynamics of individual tree collectives. High biomass carbon stocks render shifts in size distributions towards trees with larger dimensions necessary [77, 80]. The focus of our study is not on aggregated but individual tree trends. As large trees determine stand level dynamics [69], they play a major role in small-scale carbon accumulation and storage. Declines in carbon accumulation can be limited in time.

Our results highlight the contribution of old trees to the carbon capital of forests; due to their longevity they maintain and by their sustained growth simultaneously increase the carbon capital. Old trees simply might act as negative carbon senescent. This provides a key opportunity and an effective way to enhance global forest carbon storage by preventing logging old-growth tropical forests.
